# Supplementary material for: Content-rich biological network constructed by mining PubMed abstracts
Source: BMC Bioinformatics. 2004 Oct 8;5:147. doi: 10.1186/1471-2105-5-147 (PMC528731; doi:10.1186/1471-2105-5-147)
Supplement: Additional File 2 — The original results of the above study (non-essential files are deleted to keep the file size under the limit set by BMC bioinformatics). [file 1471-2105-5-147-S2.bz2 › chilibotAdditionalFile2/dip10/316ID9582097E2396/html/U2AF2_SFRS1.html]

 


 **U2AF2** and **SFRS1** 
  
Found 16 abstracts in PubMed, retrieved 10.  
 

 What does Google say? 
 PDF only 
| .edu only 

---

**Interactive relationship** (e.g. stimulation, inhibition, etc)

**Stimulatory relationship**- SF2  [ **SFRS1** ]  ASF  [ **SFRS1** ]  stimulates U2AF65  [ **U2AF2** ]  binding to the suboptimal tat polypyrimidine tract in an ESE3 dependent manner.  Ref: 11575921 J Mol Biol, 2001
- This suggests that SF2  [ **SFRS1** ]  ASF  [ **SFRS1** ]  can stimulate the recruitment of U2AF65  [ **U2AF2** ]  by an RS domain independent mechanism.  Ref: 11575921 J Mol Biol, 2001
- SF2  [ **SFRS1** ]  ASF  [ **SFRS1** ]  binds to a splicing enhancer in the third HIV 1 tat exon and stimulates U2AF  [ **U2AF2** ]  binding independently of the RS domain.  Ref: 11575921 J Mol Biol, 2001
**Neutral relationship**- Over expression of either TcSR or the human SR protein associated splicing factor splicing factor 2  [ **SFRS1** ]  ASF SF2  [ **SFRS1** ]  in wild type Schizosaccharomyces pombe, provoked an elongated phenotype similar to that of fission yeast over expressing the SR containing splicing factor Prp2, a U2AF 65  [ **U2AF2** ]  orthologue.  Ref: 12615334 Mol Biochem Parasitol, 2003

**Non-interactive relationship** (e.g. studied together, co-existance, homology, etc.)

- These findings indicate that ASF SF2  [ **SFRS1** ]  or U2AF 65  [ **U2AF2** ]  gene expression is altered during in vitro two step chemical transformation.  Ref: 10590230 Carcinogenesis, 1999
- Purified recombinant Sip1 protein, but not other RS domain containing proteins such as SC35, ASF SF2  [ **SFRS1** ] , and U2AF65  [ **U2AF2** ] , restored the splicing activity of the Sip1 immunodepleted extract.  Ref: 9447963 Mol Cell Biol, 1998
- We have further investigated the expression of five splicing regulatory factors, heterogeneous nuclear ribonucleoprotein hnRNP A1 and A2, alternative splicing factor  [ **SFRS1** ]  splicing factor 2  [ **SFRS1** ]  ASF SF2  [ **SFRS1** ] , spliceosome component of 35 kDa protein SC35 and 65 kDa subunit of U2 snRNP auxiliary factor U2AF65  [ **U2AF2** ] , using two series of clonally derived mouse fibroblast cell lines, that were SV40 large T transfectants SV T2 and NIH3T3 13C7 or c erbB2 transfectants A4.  Ref: 11295081 Oncol Rep< MedlineTA>Oncol Rep, 1998
- Gene expression of three splicing regulatory factors, alternative splicing factor  [ **SFRS1** ]  splicing factor 2  [ **SFRS1** ]  ASF SF2  [ **SFRS1** ] , heterogeneous nuclear ribonucleoprotein A2 hnRNP A2 and the 65 kDa subunit of U2 small nuclear ribonucleoprotein particles auxiliary factor U2AF 65  [ **U2AF2** ] , were examined by northern blotting in a two step chemical transformation model.  Ref: 10590230 Carcinogenesis, 1999
- Truncated SF2  [ **SFRS1** ]  ASF  [ **SFRS1** ]  protein without the RS domain binds specifically to the ESE3 and retains almost full capacity to stimulate U2AF65  [ **U2AF2** ]  binding and activate splicing.  Ref: 11575921 J Mol Biol, 2001
- The mRNA levels of the splicing factors ASF SF2  [ **SFRS1** ] , U2AF  [ **U2AF2** ]  SF3a, p54nrb and PTB was found to decrease rapidly during differentiation.  Ref: 10605820 Int J Biochem Cell Biol, 1999
- When the 3 splice site is weak, both the SF2  [ **SFRS1** ]  ASF  [ **SFRS1** ]  RS domain and U2AF  [ **U2AF2** ]  35 are required for splicing.  Ref: 11124808 Genes Dev, 2000
- This protein protein interaction profile is different from those of prototypical SR proteins SC35 and ASF SF2  [ **SFRS1** ] , both of which interact with U1 70K and U2AF35 but not with U2AF65  [ **U2AF2** ] .  Ref: 8816452 Mol Cell Biol, 1996
